# First baseline data of the Klinefelter ItaliaN Group (KING) cohort: clinical features of adult with Klinefelter syndrome in Italy

**DOI:** 10.1007/s40618-022-01816-4

**Published:** 2022-05-24

**Authors:** D. Pasquali, P. Chiodini, V. Simeon, A. Ferlin, L. Vignozzi, G. Corona, F. Lanfranco, V. Rochira, A. E. Calogero, M. Bonomi, R. Pivonello, G. Balercia, A. Pizzocaro, V. A. Giagulli, P. Salacone, A. Aversa, G. Accardo, M. Maggi, A. Lenzi, A. Isidori, C. Foresta, E. A. Jannini, A. Garolla, the KING, Klinefelter ItaliaN Group, D. Pasquali, A. Ferlin, L. Vignozzi, G. Corona, F. Lanfranco, V. Rochira, A. E. Calogero, M. Bonomi, R. Pivonello, G. Balercia, A. Pizzocaro, V. A. Giagulli, P. Salacone, A. Aversa, G. Accardo, M. Maggi, A. Lenzi, A. Isidori, C. Foresta, E. A. Jannini, A. Garolla

**Affiliations:** 1 Endocrinology Unit, Department of Advanced Medical And Surgical Sciences, University of Campania “L. Vanvitelli”, 80138 Napleszz, Italy; 2 University of Padua, Padua, Italy; 3 University of Firenze, Firenze, Italy; 4 Endocrinology Unit, Medical Department, Azienda Usl Bologna Maggiore-Bellaria Hospital, Bologna, Italy; 5 University of Turin, Turin, Italy; 6 Unit of Endocrinology, Department of Biomedical, Metabolic and Neural Sciences, University of Modena and Reggio Emilia, Modena, Italy; 7 University of Catania, Sicily, Catania Italy; 8 Dept of Endocrine and Metabolic Diseases, IRCCS Istituto Auxologico Italiano, Milan, Italy; 9 Dept. Of Biotechnology and Translational Medicine, University of Milan, Milan, Italy; 10 University Federico II, Naples, Italy; 11 University of Marche, Ancona, Italy; 12 IRCCS, Istituto Clinico Humanitas, Rozzano-Milan, Italy; 13 Interdisciplinary Department of Medicine-Section of Internal Medicine, Geriatrics, Endocrinology and Rare Diseases, School of Medicine, University of Bari Aldo Moro, Bari, Italy; 14 Outpatients Clinic of Endocrinology and Metabolic Disease, Conversano Hospital, Bari, Italy; 15 Santa Maria Goretti Hospital, Latina, Italy; 16 Department of Experimental and Clinical Medicine, University of Catanzaro, Catanzaro, Italy; 17 ASL Salerno, Salerno, Italy; 18 Department of Experimental Medicine, Sapienza University of Rome-Policlinico Umberto Hospital, Rome, Italy; 19 Department of Systems Medicine, University of Rome Tor Vergata, Rome, Italy

**Keywords:** Klinefelter syndrome, Metabolic syndrome, MetS, Testosterone, Testis, BMI, LH, FSH

## Abstract

**Background:**

Klinefelter syndrome (KS) is frustratingly under-diagnosed. KS have a broad spectrum of clinical features, making it difficult to identify.

**Objective:**

We describe KS clinical presentation in a large Italian cohort.

**Design:**

This is the first observational cohort study within a national network, the Klinefelter ItaliaN Group (KING). Primary outcomes were to describe the basic clinical features and the actual phenotype of KS in Italy. Secondary outcomes were to determine age at diagnosis and geographical distribution.

**Methods:**

We performed a basic phenotyping and evaluation of the hormonal values of 609 adult KS patients.

**Results:**

Mean age at diagnosis was 37.4 ± 13.4 years. The overall mean testicular size was 3 ml, and 2.5 ml in both testes in untreated KS group. BMI was 26.6 ± 5.8 kg/m^2^, and 25.5% of KS had metabolic syndrome (MetS). LH and FSH were increased, and mean total testosterone were 350 ± 9.1 ng/dl. A descriptive analysis showed that 329 KS patients were evaluated in Northern Italy, 76 in Central and 204 in Southern Italy. Analysis of variance demonstrated significant statistical differences (*p* < 0001) between the age at diagnosis of the three geographical groups. Compared with the expected number among male patients matched for age in Italy, only 16% of KS patients received a diagnosis.

**Conclusions:**

These data are the results of the only national database available that collects the clinical and hormonal data of the KS patients, currently referred at the KING centers. In Italy the typical KS patient is overweight, with small testes, and elevated LH and FSH. Only 25.5% of them are diagnosed with MetS. Early detection and timely treatment are mandatory.

## Introduction

Klinefelter syndrome (KS) is the most frequent chromosomal disorder associated to male infertility, occurring in 1:500 to 1:1000 live male births. Men with KS typically present tall stature, small and firm testes, and a progressive testicular secretion impairment leading to infertility and hypergonadotropic hypogonadism. Without timely replacement, testosterone deficiency can lead to delayed or incomplete puberty, gynecomastia, decreased muscle mass, decreased bone density, and a reduced amount of facial and body hair [1–6]. However, the clinical spectrum of KS patients might be more complex than classically reported in the textbooks and might benefit from an accurate re-evaluation. A wide range of clinical features and a shaded phenotype is the hallmark of KS [4]. As the disease is often overlooked, several websites were developed to facilitate the diagnosis, but their reliability is not always guaranteed and can generate confusion. However, the main problem to be considered is the high percentage of KS subjects that come to a diagnosis late in their lives (i.e., during a medical counseling for couple infertility) or, even worse, those that will never be diagnosed thus remaining unknown [4, 7]. A well-known problem is the delayed diagnosis or non-diagnosis of KS [7], due to man’s hesitancy to seek medical counseling, low awareness of KS among health professionals, and failure by health professionals to perform routine genital examinations in adult men. Furthermore, delayed diagnosis worsens the outcome of testosterone replacement therapy and assisted reproductive techniques for sperm retrieval and fertility [8]. Timely screening and intervention are needed for common and well-known associated metabolic, cardiovascular health problems and comorbidities [9–17]. There are excellent, published observational studies of KS from Denmark and the United Kingdom national health registries [18, 19]. The main strengths of registered-data epidemiology are that data already exist and there is a minimizing selection bias in the study population. Main limitations are that necessary information may be unavailable, data collection is not done by the researcher, confounder information is lacking. This leads us to the biggest risk of inaccurate conclusions of registry data the lack of uniformity of diagnostic and therapeutic approach between the reporting centers. To overcome these limits, we set up a multicenter observational cohort study collecting data from KS patients regularly attending the centers of the national network of academic or general hospitals named KING (Klinefelter ItaliaN Group), between January 2014 and June 2018. The Klinefelter Italian Group (KING) is a specify study group of the Italian Society of Andrology and Sexual Medicine (SIAMS) aimed to critically review the basic clinical features of KS in the Italian population, and to identify the actual phenotype for an early diagnosis and treatment to allow for the best quality of life. Moreover, since information about possible geographical variances in KS presentation due to different lifestyle habits or environmental factors is lacking, we decided to study it.

## Patients and methods

### Patients’ population

Multicenter observational study of 609 KS was performed among the patients regularly attending the centers of the national network of academic or general hospitals named KING (Klinefelter ItaliaN Group), between January 2014 and June 2018, after written informed consent had been obtained (the supplementary table lists the KING centers involved in the study). The participants were consecutively selected in order of appearance according to their convenient accessibility at the KING centers. In 594 KS age at diagnosis was reported. The study, accomplishing the Declaration of Helsinki, was approved by the Ethics Committee of the coordinating institution (n. 1489, 26 October 2015), and all patients or their tutors gave a written informed consent. The local Ethics Committees of each center approved the study. Anonymous patient data, referred to the time of diagnosis, before any further therapy, were collected and a clinical database was created. Inclusion criteria were (i) a verified KS karyotype (47, XXY) and (ii) a written informed consent.

### Exclusion criteria

KS patients with mosaic forms of chromosomal aneuploidy or any other structural or numerical karyotype anomaly, with AZF microdeletions, were excluded.

### Protocols

Patients and/or their parents underwent standard interviews on family history. Data collected included: (1) pubertal development (Tanner stages) and (2) testicular volume (TV) by Prader orchidometer, the physical examination was performed by the same blinded investigator in each center of the KING group. Body weight was measured (with the participants wearing underwear) to the nearest 0.1 kg, height was measured to the nearest 0.5 cm, BMI was calculated.

### Collection of samples

Venous blood (2 ml) from participants was collected by blood banks of the hospitals of the KING centers during the same period of KS recruitment. All basal blood samplings were performed before 09:00 h, after an adequate fasting period, and the low hormonal levels were always confirmed at least twice. Samples were immediately sent for analysis of Luteinizing Hormone (LH) and Follicle-Stimulating Hormone (FSH), total testosterone (T), 17ß-estradiol, sex-hormone binding globulin (SHBG), to the laboratory facility in each hospital institution of the KING groups. As we recruited patients from different centers, different methods were used. In most of the cases FSH, LH were determined by IRMA, using commercial kits (12). Serum E2 levels and total testosterone were measured by immunoassay using a commercial Kit, automated immunoassays (DiaSorin Lyiaison, Saluggia, VC, Italy). The normal male reference for the parameters studied were the following: total testosterone 249–836 ng/dl, estradiol 20–80 ng/ml. LH and FSH assays had a lower limit of detection of 0.1 IU/L and a functional sensitivity of 0.2 IU/L. The inter- or intra-assay coefficients of variation were < 5% in all assays since 2008. Free testosterone was calculated using Vermeulen Eq. (20).

### Definition of metabolic syndrome

Metabolic syndrome (MetS) was diagnosed according to the definition of the National Cholesterol Education Program (NCEP)/Adult Treatment Panel III (ATPIII), criteria (21). The study population was classified for the presence of abdominal obesity (waist circumference > 102 cm), hypertriglyceridemia (triglycerides levels equal to or > 150 mg/dl), hypercholesterolemia (HDL-cholesterol < 40 mg/dl for men with total cholesterol values > 200 mg/dl), hypertension (systolic blood pressure equal to or > 130 and/or diastolic > 85 mmHg) and impaired fasting glucose (fasting glycaemia equal to or > 110 mg/dl). Patients currently receiving antihypertensive drug, statins or oral hypoglycemic drugs were considered as having those components of the metabolic syndrome. Smoking status was defined by two dichotomous categories: smoker and non-smoker. The presence of the metabolic syndrome was defined by the coexistence of ≥ 3 among abdominal obesity, hypertriglyceridemia, hypercholesterolemia, hypertension, and impaired fasting glucose (21).

### Statistical analysis

This is multicenter observational cohort study conducted from 2014 to 2018. No longitudinal data were collected in the present study. Continuous data were represented as mean ± standard deviation (SD) or median (interquartile range—IQR). Categorical data were represented as absolute and relative frequencies. Group comparison was performed using the appropriate statistical test, in accordance with the number of groups and the type of variable (categorical, continuous normal or skewed). The population-based prevalence was estimated as the annual number of cases being alive in Italy each year during the study period (2014–2018). Incidence was estimated as the average number of diagnosed cases per million males (KS) in the background population each year during the study period. With the aim to test the association between metabolic syndrome and therapy, a logistic regression model was used. The odds ratio (OR) and its 95% confidence interval (95%CI) was calculated. The model was further adjusted for related clinical variable. All statistical analysis was performed using STATA v16 (StataCorp. 2019. Release 16. College Station, TX: StataCorp LLC.). *p* Value less than 0.05 was considered statistically significant.

## Results

### Characteristics

In our multicenter, observational study of 609 KS, the mean testicular size was 3 ml in each testis. Volume measures of both testes were evaluated using the Prader orchidometer and the physical examination was performed by the same blinded investigator in each center of the KING group. BMI was 26.6 ± 5.8 kg/m^2^ and TESE was performed in 68 patients out of 402 (16.9%). Twenty five percent (25.5%) of the patients with KS developed MetS (Table 1).

**Table 1:** Clinical characteristics of Klinefelter syndrome patients in Italy

*n*	609
Age (years)	
Median (IQR)	36 (28–46)
Mean (sd)	37.4 (13.4)
BMI	
Mean (sd)	26.6 (5.8)
Test DX, size (prader orchidometer)	
Median (IQR)	3 (2–3.85)
Test SX, size (prader orchidometer)	
Median (IQR)	3 (2–4)
TESE	
No *n* (%)	334 (83%)
Yes *n* (%)	68 (16.9%)
MetS	
No (%)	436 (74.4%)
Yes (%)	150 (25.5%)

### Sex hormones

**Fig. 1:**
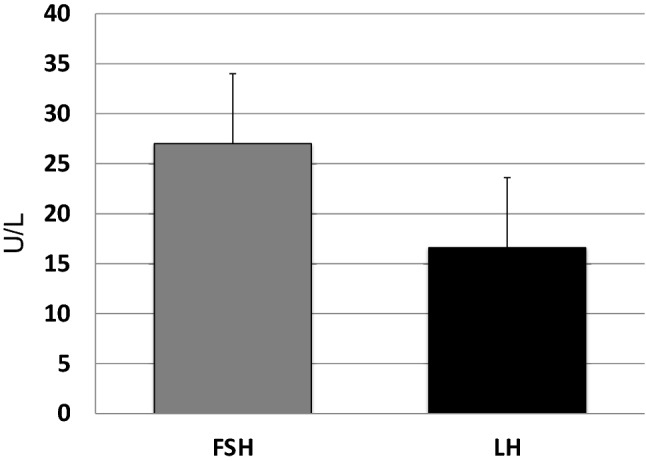
FSH and LH plasma levels in Klinefelter syndrome patients

**Fig. 2:**
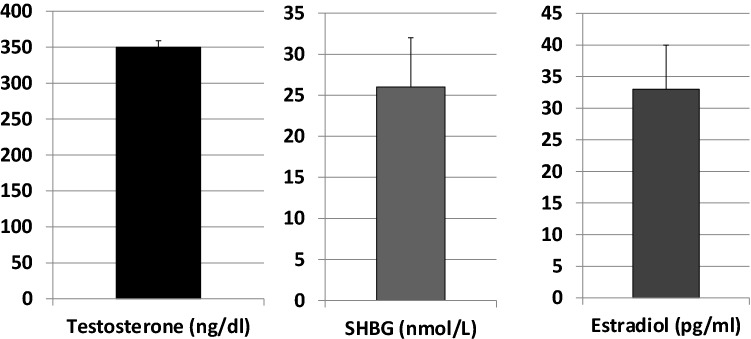
Total Testosterone, SHBG and 17beta estradiol in Klinefelter syndrome patients

Overall, mean LH and FSH levels were 16.6 IU/L (median and IQR: 8.8–22.5) and 28.5 IU/L (median and IQR: 17.5–39), respectively (Fig. 1) and mean T was 350 ± 9.1 SD ng/dl. LH/T ratio remains high despite normal T levels. 214 out of 594 patients (40%) had total testosterone below the normal limit (271–965 ng/ml) 17ß-estradiol was 26 pg/ml (median and IQR: 19.3–33) and SHBG 33 nmol/l (median and IQR: 21.9–41.1). Calculated free testosterone was 70 pg/ml (Fig. 2).

### Age at diagnosis

A total of 609 patients were diagnosed to have KS during the period of observation of this study (from 2014 to 2018). The mean age at diagnosis was 37.4 ± 13.4 years (median IQR 28–46). There was no change in age at diagnosis during 2014–2018 among all KS. Diagnosis of KS before 18 years of age was made only in 46 out of 594 patients (7.7%) (Table1).

### Geographical distribution of Klinefelter syndrome in Italy

**Fig. 3:**
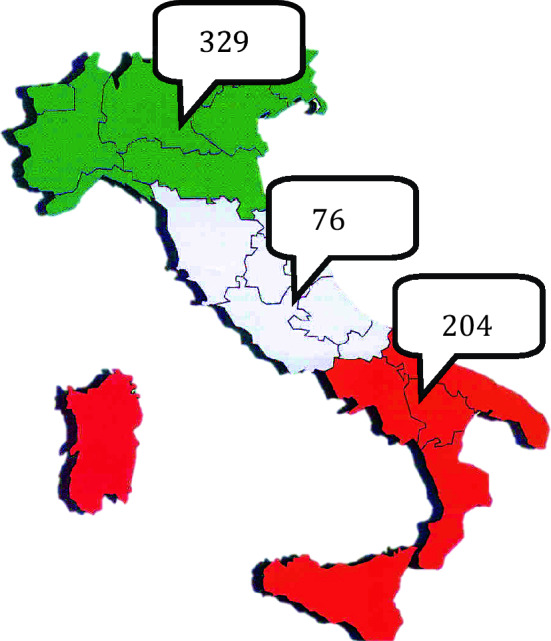
Geographical distribution of Klinefelter syndrome patients referred to KING centers in Italy

**Fig. 4:**
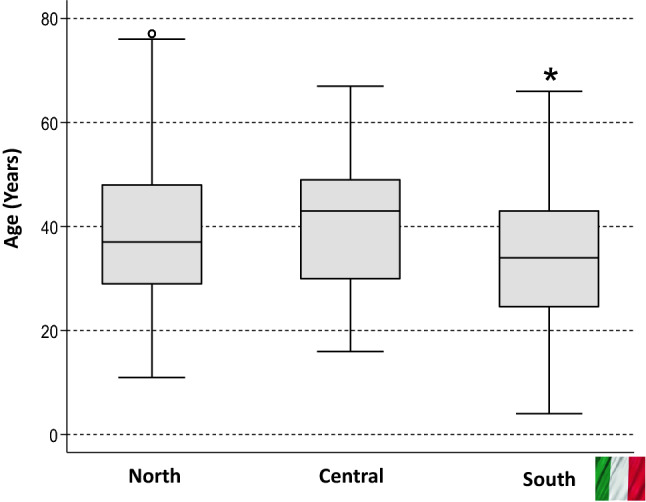
Geographical differences of the age at diagnosis in Italy, *p < 0000 vs. North and Central Italy

Mean male population in Italy during 2014–2018 was 29.463,164 and only 609 KS were referred to KING centers. Compared to the expected population-based prevalence, 1 every 1000 males in the general population, only 2% (609 out of 29.000) were identified. A descriptive analysis was performed and showed that 329 KS were referred to KING centers in Northern Italy, 76 and 204 KS to KING facilities in Central and Southern Italy, respectively (Fig. 3). Analysis of variance showed significant statistical differences (*p* < 0.0001) between KS age at diagnosis in the three geographical areas. In particular, age at diagnosis was significantly lower in Southern Italy (33.3 ± 13 SD) compared to Central and Northern Italy (40.2 ± 12.5 SD and 39.2 ± 13.3 SD) (Fig. 4).

## Discussion

These are the first baseline data collected by the KING group database from 2014 to 2018. According to what is currently known, this is the first and only national database that collects the clinical and hormonal data of the KS adult patients currently in treatment at the accredited centers. The typical man with KS in Italy is overweight, with small testes and presents increased plasma levels of FSH and LH, despite normal T levels. The results of this study confirmed that KS patients present a median T level in the low-normal range, with reciprocally increased levels of LH and FSH clearly showing that these KS patients are hypogonadal, in line with literature data [1, 22, 23]. Moreover, our results help support the evidence that KS is a highly under-diagnosed condition, also in Italy.

A novelty of the present study is that the frequency of MetS in patients with KS referred to the highly specialized Italian network KING is surprisingly lower than reported elsewhere [24, 25]. Other reports showed epidemiological and clinical evidence for a fourfold increased risk of diabetes and MetS in KS [26]. The reduced percentage of MetS in our patients, 25% compared to 44% described in the literature [24] and 34.3% from Ishikawa and colleagues cannot be related to the participants’ age that was similar [25]. The mean BMI of Italian KS was ~ 27 kg/m^2^, showing that the patients were overweight, and it was like previous reports [24]. The reason of the different prevalence of MetS is not clear. Differences in dietary habit and food consumption could be relevant. Recent studies from the literature underlie the relevance of testosterone treatment in adult KS men leading to favorable changes in body composition with reduction in fat mass, including abdominal fat mass, but does not change glucose homeostasis [26, 27]. It is known that low testosterone levels are common and associated with insulin resistance in men with diabetes [28–30]. In literature, reduced testosterone concentrations (< 12 nmol/L) are found in variable percentages (65–85%) of adults with KS (23). Elevated gonadotropin levels are always present even in those KS subjects presenting testosterone levels still in the normal range [1, 4, 23]. Due to heterogeneous serum testosterone concentrations in KS, the adequate threshold below which serum testosterone should be considered insufficient in these subjects is lacking [31]. Controlled studies showing a different age-related hypogonadism in patients with KS are not available. The impact of the genetic alteration on testis function is clear [32], while the overall lower incidence of MetS seen in the cohort could highlights the importance of the dietary habits [33–35]. Moreover, to better understand the complexity of KS phenotype, more research is needed to identify all the unknown effectors of the chromosomal imbalance.

The mean age at diagnosis of KS in Italy was 37.4 ± 13.4 years, like other reports (38.7 and 33.6 ± 5.3 years, respectively) [24, 25], while is lower in Australia [36]. We did not find any change in the age of diagnosis during 2014–2018 among all KS. Diagnosis of KS before 18 years of age was made only in 46 out of 609 patients (7.5%). This data can be partially explained by the fact that the KING centers are responsible for the care mainly of adult men. Transition from pediatric to adult care for KS patients should be improved, and a more accurate evaluation and awareness between health care professional is required, considering that boys with 47, XXY are difficult to identify during childhood [37, 38]. The comparison with data from prenatal testing and paediatric clinics shows that patients are underdiagnosed. In addition, some interesting age differences at diagnosis emerged between the three geographical areas. The mean age at diagnosis was significantly lower in Southern Italy compared to Central and Northern Italy. We can speculate that among KING centers in Northern Italy there are well-established fertility clinics, nationwide reference for cryopreservation and testicular sperm extraction techniques; therefore, the age of the patients referring to them could be higher [39]. Data of this study come from a database of all centers of the national network of academic or general hospitals named KING (Klinefelter ItaliaN Group), thus geographical distribution is calculated on the Italian population.

Finally, our data indicate the only generally shared clinical manifestation is the low bitesticular volume and the increased serum FSH and LH levels. The most surprising fact is that the KS patients in our cohort present MetS in a lower percentage compared to other finding.

In conclusion, the results of the first baseline data of the Klinefelter Italian Group (KING) cohort study have clearly shown that although KS has been known for over 70 years, there is still much work to be done for early diagnosis and proper management of these patients. The KING database will allow the introduction of data on the same patient over time, compared to register studies. Longitudinal studies of this cohort will give us important clues on the management and follow-up of KS, also in terms of quality of life and life expectancy. However, still too many patients escape the diagnosis and that delay in identifying the presence of KS can have a detrimental effect on the quality-of-life of these men, impeding the start of replacement treatment and the evaluation on the associated disorders [40]. Therefore, standardized guidelines for proper management in the light of an accurate reassessment of all systemic comorbidities are strongly needed [41]. A close cooperation between pediatricians and endocrinologists is necessary to ensure a successful transition, to reduce the undiagnosed rate together with a better awareness in diagnosing KS subjects at prepubertal age.
